# Beta-Blocker-Induced Erythrodermic Psoriasis: A Case Report

**DOI:** 10.7759/cureus.29809

**Published:** 2022-10-01

**Authors:** Diana Voloshyna, Saad Ehsan Ullah, Nusrat Jahan, Sai Sreya Yadlapalli, Mohammad Munim Zahoor, Yumna Shams, Meenakshi Sathish, Qudsia I Sandhu, Farhan Saleem

**Affiliations:** 1 School of Medicine, University of Michigan, Ann Arbor, USA; 2 Internal Medicine, Shaikh Zayed Hospital, Lahore, PAK; 3 Internal Medicine, Dhaka Medical College, Dhaka, BGD; 4 Medical Student, Spartan Health Sciences University, Vieux Fort, LCA; 5 Internal Medicine, Ghurki Trust Teaching Hospital, Lahore, PAK; 6 Internal Medicine, Dow University of Health Sciences, Dow International Medical College, Karachi, PAK; 7 Surgery, Caribbean Medical University School of Medicine, Chicago, USA; 8 Medicine, Ghazi Khan Medical College, Dera Ghazi Khan, PAK; 9 Orthopaedic Surgery, Lahore General Hospital, Lahore, PAK

**Keywords:** beta-blocker-induced psoriasis, beta-blocker, case report, erythrodermic psoriasis, drug-induced psoriasis, psoriasis

## Abstract

Beta-blockers are well-known for their wide range of therapeutic applications, particularly in patients with cardiac diseases. Physicians worldwide are aware of their potential side effects, including hypoglycemia, dizziness, slow heart rate, fatigue, and heart block. We report a case of erythrodermic psoriasis caused by beta-blockers in a 61-year-old woman with no prior history of the skin condition. The diagnosis was made based on the characteristic histopathological picture and a Naranjo score of 6. She was administered 15 mg of methotrexate weekly and received supportive care. She recovered completely within two months and exhibited no recurrence of symptoms.

## Introduction

Psoriasis is a common skin disorder prevalent worldwide. Approximately 4.6% of the population in the United States is affected by psoriasis, and 2.25% of these patients may experience erythrodermic psoriasis (EP) [1]. EP is a chronic form of plaque psoriasis that causes the formation of inflammatory erythematous plaques on the skin and edema. The diagnostic criteria for EP require 75% of the affected body surface area. The complications of EP include multisystem organ failure that can lead to mortality [2]. EP is mainly exacerbated by the use of certain medications, mainly beta-blockers, immunosuppressive drugs, and lithium, as well as in patients with a family history of psoriasis. Other risk factors include medication withdrawal and emotional stress [3]. There are many reported cases of drug-induced EP. Patients with a history of psoriasis are at a major risk of developing EP. The diagnosis of drug-related psoriasis can be done using the Naranjo scale, which shows the probability of adverse drug reactions [4]. We present the case of a 61-year-old patient with beta-blocker-induced EP.

## Case presentation

A 61-year-old woman with a medical history of coronary artery disease (CAD) presented to the outpatient department of a tertiary hospital with recent onset of an erythematous and scaly rash over her hands and feet, which had progressed to the trunk. Recently, the cardiac care unit diagnosed her with stable angina and prescribed bisoprolol as her only medication. Her initial bisoprolol dosage of 2.5 mg was well tolerated. Subsequently, it was adjusted to 10 mg twice daily. The patient continued to take the bisoprolol as prescribed and had no complaints at her four-week follow-up. However, after five weeks of taking 10 mg bisoprolol twice daily, she developed an erythematous scaly rash. The patient had no prior experience with trauma or any kind of skin lesion of this nature. There was no history of atopy or seasonal allergies in her family. There was no prior history of skin lesions in her family.

**Figure 1:**
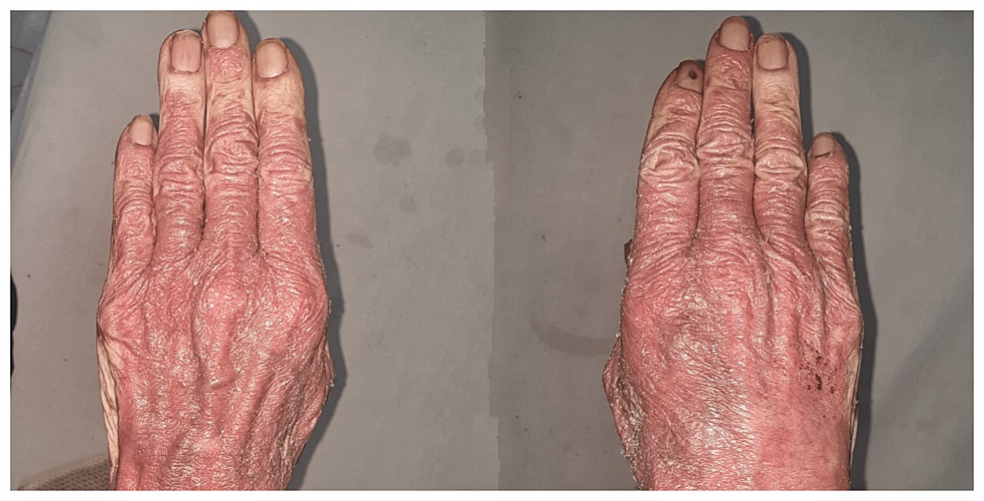
Dorsal aspect of the hands showing erythrodermic psoriasis.

**Figure 2:**
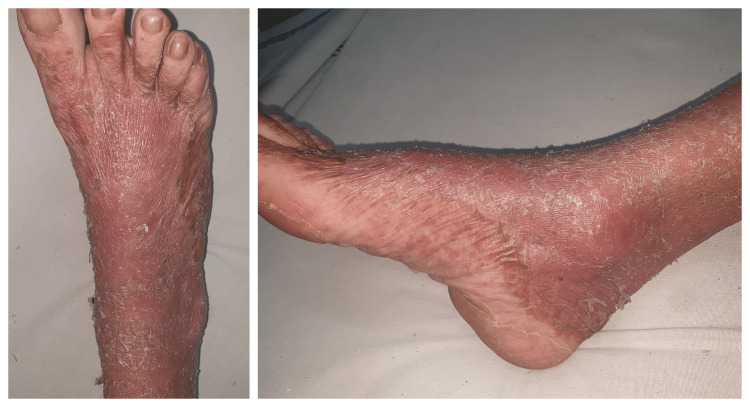
Dorsal and lateral aspect of the right foot showing erythrodermic psoriasis.

Her skin examination revealed erythematous and scaly lesions, particularly on the dorsal surface of her hands (Figure 1) and feet (Figure 2).

Her face was spared entirely. Her nails, hair, and mucosal surfaces were normal. Her systemic examination was unremarkable, and her vitals were stable. There was no evidence of dehydration. Her lab results, which included a complete blood count (CBC), liver function test (LFT), renal function test (RFT), eosinophils, immunoglobulin E (IgE), and antinuclear antibodies (ANAs), were unremarkable, which excluded any atopic or autoimmune condition. A dermatologist was consulted who diagnosed it as beta-blocker-induced EP and recommended a biopsy to confirm the diagnosis. Over the course of four days, the beta-blocker was gradually tapered off and replaced with a calcium channel blocker. She was prescribed topical medications, oatmeal baths, and supportive care along with an oral dose of methotrexate 15 mg weekly. Her biopsy revealed psoriasiform dermatitis with parakeratosis, the absence of the granular layer, elongated rete ridges, suprapapillary thinning of the epidermis, focal lymphocytic infiltrates, and marked dilation and coiling of vessels within the papillary dermis, all of which were consistent with a drug-induced EP.

Her lesions diminished following the discontinuation of beta blockers and resolved completely within two months. She never experienced a recurrence of these lesions during this time and refused a retest of beta-blocker when requested at her six-month follow-up. Her biopsy results and the rating of 6 on the Naranjo scale [4] (Table 1) led to the conclusive diagnosis of beta blocker-induced EP.

**Table 1:** Naranjo scale of adverse reaction.

Questions	Yes	No	Do not know	Patient
1. Are there previous conclusive reports on this reaction?	1	0	0	1
2. Did the adverse event appear after the suspected drug was administered?	2	-1	0	2
3. Did the adverse reaction improve when the drug was discontinued or a specific antagonist was administered?	1	0	0	1
4. Did the adverse event reappear when the drug was re‐administered?	2	-1	0	0
5. Are there alternative causes (other than the drug) that could on their own have caused the reaction?	-1	2	0	0
6. Did the reaction reappear when a placebo was given?	-1	1	0	1
7. Was the drug detected in blood (or other fluids) in concentrations known to be toxic?	1	0	0	0
8. Was the reaction more severe when the dose was increased or less severe when the dose was decreased?	1	0	0	0
9. Did the patient have a similar reaction to the same or similar drugs in any previous exposure?	1	0	0	0
10. Was the adverse event confirmed by any objective evidence?	1	0	0	1
Total score:				6

## Discussion

EP is characterized by erythema that affects 75-90% of the body surface area. Patients with EP exhibit symptoms such as fever, dehydration, chills, pruritis, asthenia, arthralgia, and lymphadenopathy due to extensive cutaneous involvement. EP can be triggered by infections, systemic corticosteroids, withdrawal of medications, previous illness, or extreme emotional stress [3]. Other etiological factors include family history, hypersensitivity, HIV infection, aggravated dermatosis, and cutaneous and hematologic cancers. Approximately 30% of EP cases have an unknown etiology [2].

Numerous case reports have described the exacerbation or induction of EP by the administration of certain drugs. Studies have reported strong associations between beta-blockers, terbinafine, lithium, and antimalarial drugs (hydroxychloroquine, imiquimod, and interferons) and EP. The histopathological and clinical characteristics of drug-induced psoriasis differ from those of nondrug-related psoriasis, with a longer latency period for drug-induced psoriasis compared to other forms [4]. The most commonly prescribed medications for the treatment of cardiac and noncardiac disorders are beta-blockers. They treat a range of conditions, including arrhythmias, heart failure, CAD, hypertension, thyrotoxicosis, anxiety, and migraines. Beta-blockers have been associated with various adverse effects, including fatigue, sexual dysfunction, and gastrointestinal distress [5]. Recent research indicates that beta-blockers are the primary agents responsible for the development of EP in adults. It frequently occurs within 12 months of beta-blocker administration [4].

A study on the association between hypertension and psoriasis found that only beta-blockers increased the risk of psoriasis in hypertensive patients receiving long-term drug therapy [6]. In another study, a 66-year-old woman with a history of psoriasis was hospitalized after developing a severe rash after two months of regular metoprolol intake for atrial fibrillation. She had diffuse erythroderma and widespread scaling. The evaluation demonstrated that metoprolol was the cause of her psoriasis. Precaution should be taken before prescribing beta-blockers to patients who are at risk or have a history of psoriasis [7]. There are no well-described case studies of psoriasis in children caused by beta-blockers, with the exception of an 18-month-old girl who developed a psoriasiform diaper rash following oral treatment with propranolol [8].

The pathophysiology underlying the induction of psoriasis by beta-blockers remains unclear. According to studies, beta-blockers reduce intracellular calcium levels and accelerate keratinocyte proliferation [5]. This is because the cyclic adenosine monophosphate messenger system is blocked. The increase in keratinocytes and polymorphonuclear leukocytes caused by beta-blockers triggers inflammation, resulting in the development of psoriasis [9]. It is essential to identify the cause of psoriasis to develop an optimal treatment plan for patients. However, identifying psoriasis caused by medications is challenging. The Naranjo scale for adverse drug reactions is an appropriate instrument for distinguishing drug-induced psoriasis [4]. Our patient scored a 6 on the Naranjo scale, falling in the probable category. In the majority of patients, psoriasis is diagnosed by physical examination and morphological analysis. Biopsies can also be performed for diagnostic purposes, but they are uncommon, particularly in children [10].

The first and most important step after a diagnosis of EP due to a drug is to discontinue and replace the offending drug [4]. Lesions on the skin are treatable with topical corticosteroids, vitamin D analogs, and adequate hydration. There is a risk of infection that should be managed appropriately, and immunosuppressants should be administered if symptoms are not under control [2]. To control drug-induced psoriasis, immunosuppressants such as cyclosporine, infliximab, and methotrexate are frequently used [2,4,7,9]. Other treatment options for patients with long-term, stable EP include phototherapy. As photosensitization can increase the likelihood of Koebnerization, it is contraindicated in acute EP. It can be used as a supplementary treatment for EP [3].

## Conclusions

This case illustrates how the use of beta-blockers, a common class of medications, can trigger the onset of a rare form of psoriasis in a patient who has never had psoriasis before. When a patient develops a rash following the initiation of a medication, it is important to consider EP in the differential diagnosis. In cases where drug-induced psoriasis is suspected, the drug should be stopped immediately.

## References

[1] Foss MG, Nyckowski T, Steffes W (2021). Erythrodermic psoriasis exacerbated by bupropion. Cureus.

[2] Rendo M, Boster J, Dalton SR, Yun H (2019). An uncommon presentation of erythrodermic psoriasis in a patient without a history of psoriasis. Cureus.

[3] Lo Y, Tsai TF (2021). Updates on the treatment of erythrodermic psoriasis. Psoriasis (Auckl).

[4] Balak DM, Hajdarbegovic E (2017). Drug-induced psoriasis: clinical perspectives. Psoriasis (Auckl).

[5] Awad VM, Sakhamuru S, Kambampati S, Wasim S, Malik BH (2020). Mechanisms of beta-blocker induced psoriasis, and psoriasis de novo at the cellular level. Cureus.

[6] Wu S, Han J, Li WQ, Qureshi AA (2014). Hypertension, antihypertensive medication use, and risk of psoriasis. JAMA Dermatol.

[7] Doyon JB, Liu KJ, Berman RA (2017). Metoprolol-induced total body erythroderma. J Gen Intern Med.

[8] Baggio R, Le Treut C, Darrieux L, Vareliette A, Safa G (2016). Psoriasiform diaper rash possibly induced by oral propranolol in an 18-month-old girl with infantile hemangioma. Case Rep Dermatol.

[9] Waqar S, Sarkar PK (2009). Exacerbation of psoriasis with beta-blocker therapy. CMAJ.

[10] Bronckers IM, Paller AS, van Geel MJ, van de Kerkhof PC, Seyger MM (2015). Psoriasis in children and adolescents: diagnosis, management and comorbidities. Paediatr Drugs.

